# A ‘High Risk’ Lifestyle Pattern Is Associated with Metabolic Syndrome among Qatari Women of Reproductive Age: A Cross-Sectional National Study

**DOI:** 10.3390/ijms17060698

**Published:** 2016-06-02

**Authors:** Mohammed Al Thani, Al Anoud Al Thani, Walaa Al-Chetachi, Badria Al Malki, Shamseldin A. H. Khalifa, Ahmad Haj Bakri, Nahla Hwalla, Lara Nasreddine, Farah Naja

**Affiliations:** 1 Public Health Department, Ministry of Public Health, Doha, Al Rumaila West, 42 Doha, Qatar; malthani@sch.gov.qa; 2 Health Promotion and Non Communicable Disease Prevention Division, Ministry of Public Health, Doha, Al Rumaila West, 42 Doha, Qatar; aalthani@sch.gov.qa (A.A.A.T.); walchetachi@sch.gov.qa (W.A.-C.); balmalki@phcc.gov.qa (B.A.M.); skhalifa1@sch.gov.qa (S.A.H.K.); abakri@sch.gov.qa (A.H.B.); 3 Nutrition and Food Sciences Department, Faculty of Agriculture and Food Sciences, American University of Beirut, P.O. BOX 11-0.236, Riad El Solh, 11072020 Beirut, Lebanon; nahla@aub.edu.lb

**Keywords:** lifestyle patterns, metabolic syndrome, factor analysis, women of childbearing age, nutritional epidemiology

## Abstract

This study investigated the effect of lifestyle patterns, as a combination of diet, physical activity and smoking, on Metabolic Syndrome (MetS) among Qatari women of childbearing age (*n* = 418), a population group particularly vulnerable to the health sequela of this syndrome. Using data from the National WHO STEPwise survey conducted in Qatar in 2012, Principal Component Factor Analysis was performed to derive lifestyle patterns with survey variables related to the frequency of consumption of 13 foods/food groups, physical activity levels, and smoking status. MetS was diagnosed using ATPIII criteria. Three lifestyle patterns were identified: ‘High Risk’ pattern, characterized by intakes of fast foods, sweets and sugar sweetened beverages, in addition to lower levels of physical activity and higher smoking prevalence; ‘Prudent’ pattern, driven mainly by higher intakes of fruits, vegetables, fish, and whole grains; and ‘Traditional’ pattern which included beans, meat, dairy products, and a low prevalence of smoking. Among these three lifestyle patterns, only the ‘High Risk’ was associated with MetS, whereby subjects belonging to the third tertile of this pattern’s score had 2.5 times the odds of MetS compared to those belonging to the first tertile. The findings of this study demonstrated the synergy among high risk behaviors among Qatari women in increasing the odds of MetS; the latter being a major risk factor for cardiovascular diseases.

## 1. Introduction

Diet, physical activity and smoking are postulated as important lifestyle modulators of the risk of many lifestyle-related diseases including Non communicable diseases (NCDs). Though studies of the association between each of those factors with various NCDs have greatly advanced our understanding of their increasing prevalence, these lifestyle factors often exert their effects in a synergistic manner which would not be captured when studying each individually [1]. In many instances, to account for the interaction and synergistic effects between these factors, the traditional approach, based on single lifestyle factor, resorted to sophisticated statistical techniques such as multiple linear and logistic regression models. However, with many correlated exogenous variables, these regression models may be unstable and could result in large confidence intervals for the regression parameters [2]. Furthermore, when a large number of variables are entered in a regression model, it is possible to obtain significant association simply by chance [3]. Recently, a novel approach in nutritional epidemiology has been proposed whereby lifestyle patterns, as a combination of diet, physical activity and smoking are examined in relation to diseases such as diabetes [4]; obesity [5] and hypertension [1,6]. Not only does this approach account for the collinearity between risk factors, it also captures the complexity of real lifestyle effect on disease risk and leads to a better understanding of high-risk behaviors. In turn, this approach allows for developing more culturally sensitive public health recommendations and interventions strategies that can be readily interpreted and followed by the general population.

A common denominator associated with many NCDs is the metabolic syndrome (MetS), defined as a cluster of metabolic abnormalities including insulin resistance, abdominal obesity, abnormal lipid profile, and elevated blood pressure [7,8]. According to the International Diabetes Federation (IDF), an estimated 25% of the world’s adult population have the MetS [9]. The prevalence of this syndrome displays a wide geographical variation, with rates steadily increasing in many developing countries [10], particularly among countries of the Gulf Cooperation Council (GCC). A systematic review of the epidemiology of MetS in GCC reported prevalence rates of 20.7% to 37.2% among men and 32.1% to 42.7% among women. These rates are 10 to 15% higher compared to those found in Western countries [11] . In Qatar, although national prevalence data on MetS is not available, findings of the World Health Survey (WHS) in 2006 and later the WHO (World Health Organization) STEPwise survey in 2012 indicated significant increase in prevalence of lifestyle related NCDs such as obesity, diabetes and hypertension [12,13], all of which are strongly associated with MetS [7,8].

Of particular concern is MetS among women of reproductive age which has negative effects on fertility, pregnancy outcomes, and the overall health of both mother and child. The effect of MetS on fertility is further augmented by the concomitant presence of obesity and polycystic ovary syndrome, both of which are linked to lower oocyte quality, infertility and compromised pregnancy outcome [11]. In addition, the metabolic stress of pregnancy, including insulin insensitivity and increased blood triglyceride, LDL and cholesterol concentrations, is further exacerbated among pregnant women with preexisting MetS [12,14]. In fact, studies have shown that the presence of MetS before pregnancy increases significantly the odds of pregnancy complications, including gestational diabetes, gestational hypertension, and preeclampsia [12,13,15]. These complications place both the mother and the child at immediate and long term health risks [12,13], especially with accumulating evidence showing a significant role for lifestyle factors during the first 1000 days in affecting disease risk later in life [16].

The considerable prevalence of the MetS coupled to its significant pathophysiologic and health effects, particularly among women of reproductive age, necessitates the search for mitigating factors and strategies to address it. Lifestyle factors, including poor dietary habits, physical inactivity and smoking are often reported in association with MetS [17]. In light of the recent advances in nutritional epidemiology, the aim of the study was to examine the effect of lifestyle patterns, as a combination of diet, physical activity and smoking, on the odds of MetS, using data from the National WHO STEPwise survey conducted in Qatar in year 2012. The specific objectives include (1) examining the prevalence of MetS and its components among Qatari women of reproductive age; (2) identification of the various lifestyle patterns prevalent in this age group; and (3) investigating the association of these patterns with MetS. Findings of this study will be foundational in the formulation of culturally sensitive, evidence-based intervention strategies and programs for the prevention of MetS and its related diseases among women of child-bearing age in Qatar.

## 2. Results

**Figure 1:**
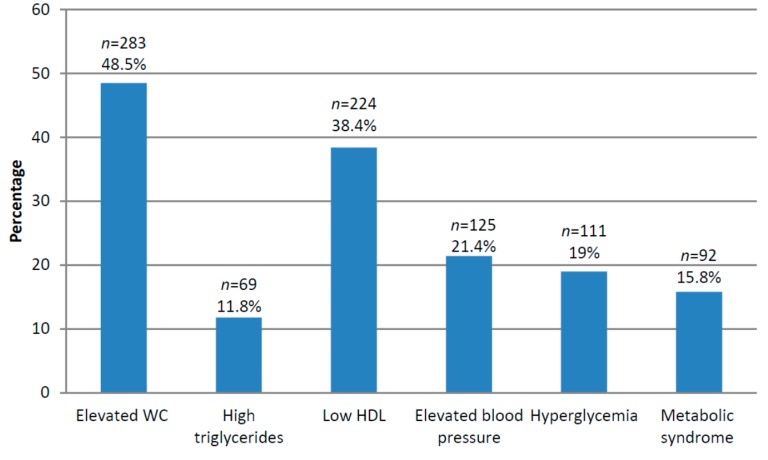
Prevalence of Metabolic syndrome and its abnormalities in the study sample * (*n* = 584). The following cut points were used: (1) WC over 88 cm; (2) fasting triglyceride (TG) level over 150 mg/dL (Or on drug treatment for elevated triglycerides); (3) fasting high-density lipoprotein (HDL) cholesterol level less 50 mg/dL (Or on drug treatment for reduced HDL-C); (4) blood pressure ≥ 130/85 mmHg (or on antihypertensive drug treatment in a patient with a history of hypertension); and (5) fasting blood sugar ≥ 100 mg/dL (or on drug treatment for elevated glucose). MetS was defined by the ATPIII criteria, whereby MetS is present if a woman had three or more of the aforementioned five criteria.

The prevalence estimates of MetS and its components among Qatari women of childbearing age are presented in Figure 1. Prevalence of MetS was 15.8%, with elevated Waist Circumference (WC) as the most common component (48.5%), followed by low High Density Lipoprotein (HDL) levels (38.4%), elevated blood pressure (21.4%), hyperglycemia (19%), and high triglyceride (TG) levels (11.8%) (Figure 1).

In order to avoid reverse causality, the associations between lifestyle factors and MetS were examined in the study population excluding women with a known diagnosis of hypertension, diabetes or any other health condition that may affect their dietary intake (*n* = 99). In addition, subjects were excluded if they had missing or incomplete dietary intake data (*n* = 67). Accordingly, associations of lifestyle patterns and MetS were carried out on 418 subjects, whose characteristics are described in Table 1. Mean age of participants was 30.89 ± 6.96 years with 51.9% belonging to 25–34 year-age group. A significant proportion of participants had a positive family history of diabetes (70.3%) and family history of hypertension (65.8%). Prevalence of smoking was low in the study sample (2.2%) and 24.6% belonged to the ‘high’ category of physical activity. Of participants, 36.1% were obese (Body Mass Index (BMI ≥30 kg/m^2^) (Table 1). The prevalence estimates of MetS and its components among the 418 study participants were as follows: MetS: 10.1%; elevated WC: 44.5%; elevated blood pressure: 13.4%; hyperglycemia: 13.9%; low HDL: 37.6% and high TG: 11.5% (data not shown). Table 1 also describes the association of various sociodemographic, lifestyle and anthropometric characteristics with MetS among study participant (*n* = 418). Prevalence of MetS increased with increasing age (18–24 years: 2.8%; 25–34 years: 9.22%; 35–45 years: 14.7%). Every additional year in age incurred a 9% increase in the odds of MetS (Odds Ratio (OR): 1.09, 95% CI: 1.04–1.14) with subjects aged between 35 and 45 years having 5.83 times the odds of MetS as compared to those aged 18–24 years. Having a positive family history of diabetes also increased the odds of MetS to 2.45, 95% CI: 1.03–50.82). Among lifestyle factors, current smoking was significantly associated with the odds of MetS in the study population (OR: 5.46, 95% CI: 1.40–21.25). A unit increase in BMI increased the odds of MetS by 11%, with obese participants (BMI ≥ 30 kg/m^2^) having 10 times more odds to be diagnosed with the MetS compared to those with BMI < 30 kg/m^2^ (Table 1).

**Figure 2:**
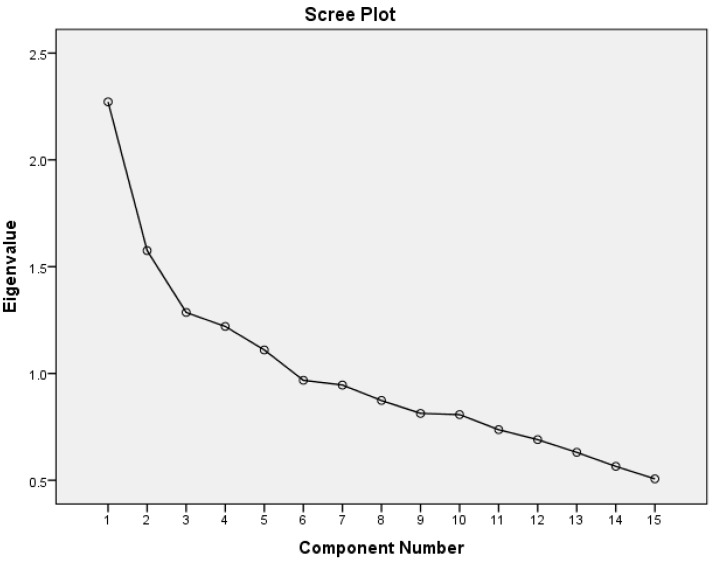
Scree plot of the PCFA using the 15 dietary and lifestyle characteristics of women participating in the study (*n* = 418).

The results of the Principal Component Factor Analysis (PCFA) conducted using lifestyle characteristics including dietary intake data (13 foods/food groups), smoking and physical activity are presented in Figure 2 and Table 2.

Figure 2 displays the screen plot showing the eigenvalues for the 15 components derived from the PCFA. Five components had an eigenvalue greater than 1. The inflection point on the plot was at the third component (inclusive), hence three components were retained and their factor loading are shown in Table 2. Together these patterns explained 34.22% of the total variance in the data. The first lifestyle pattern was characterized by intake of fast foods, sweetened beverages, sweets and refined grains, in addition to smoking. Physical activity loaded negatively on this pattern, indicating its association with low levels of physical activity. The first identified pattern in this study was thus labeled ‘High risk’ lifestyle pattern. Variables loading higher than 0.3 on the second pattern were fruits and vegetables, natural juices and fish. Whole grains had a border line loading of 0.3. Physical activity and smoking did not load on this pattern. Given its dietary characteristics, the second lifestyle pattern was labeled ‘Prudent’. The third pattern derived in this study was labeled ‘Traditional’ pattern as it had many elements of traditional Qatari lifestyle such as high intakes of beans, meat, dairy products, as well as refined grains. Smoking loaded negatively on this pattern, indicating a low prevalence of smoking associated with this pattern (Table 2). Physical activity did not load on this pattern.

The associations between the identified lifestyle patterns and their components was further examined by dividing each pattern score into 1st, 2nd and 3rd tertiles corresponding to low, middle and high levels of adherence respectively. Dietary intake, physical activity, and smoking characteristics of study participants were compared between the first and third tertiles of each of the patterns’ scores (Table 3). Subjects belonging to the third tertile of the ‘High Risk’ pattern had significantly higher intakes of fast foods, sweetened beverages, sweets, refined grains, meat and poultry, in addition to lower levels of physical activity and higher smoking prevalence. As for the ‘Prudent’ pattern, compared to the first tertile, subjects belonging to the third tertile has significantly higher frequencies of consumption of whole grains, fruits, vegetables, natural juices, beans and fish and sea foods as well as lower frequencies of consumption of refined grains. No significant differences were noted for physical activity and smoking between the first and third tertiles of this pattern’s scores. With regard to the ‘Traditional’ pattern, frequencies of consumption of refined grains, fruits, natural juices, beans, meat and dairy products were significantly higher while prevalence of smoking and physical activity level significantly lower in the third compared to the first tertile of this pattern’s scores (Table 3).

The independent associations of the derived lifestyle patterns tertiles with MetS and its components were examined using multivariate logistic regression. The resulting OR and their 95% CI are presented in Table 4. These models were adjusted for age, education, marital status, job type, parental consanguinity, a family history of diabetes, family history of high blood pressure, passive smoking, and number of meals not eaten at home. Compared to the first tertile, subjects belonging to the third tertile of the ‘High Risk’ pattern had 96% increase in the odd of hypertension (OR: 1.96, 95% CI: 1.02–4.70) and a 2.5 folds increase in the odds of MetS (OR: 2.47, 95% CI: 1.04–5.39). The ‘Prudent’ and the ‘Traditional’ patterns did not show any association with MetS and any of its components (Table 4).

## 3. Discussion

Recent advances in nutritional epidemiology has promoted transcending reductionism in nutrition research towards adopting ‘holism’ for studying health conditions with complex etiologies such as the MetS [20]. Instead of focusing on one behavioral factor alone (diet or physical activity or smoking) and its linear relationship with MetS, the holistic approach focuses on the whole lifestyle pattern and the circular causalities among risk factors. As such, a lifestyle pattern is not viewed as the sum of diet, physical activity and smoking but is rather considered as the dynamic interaction between them. This implies that a lifestyle pattern defined through diet, physical activity and smoking characteristics may exert an effect on health exceeding that of any of its parts and could lead to more ‘real life’ associations and implications. Hence the significance of this study is in combining diet, physical activity and smoking characteristics into lifestyle patterns and studying the effect of these patterns on disease risk.

In this study, among the three identified lifestyle patterns, adherence to the ‘High Risk’ pattern, characterized by unhealthy diet, low physical activity and smoking, was associated with a higher odd of MetS. This finding is in line with that of a previous investigation of the impact of combined lifestyle factors on MetS in Korean men [21], whereby subjects who had three or more of poor lifestyle factors (smoking, high risk alcohol use, low physical activity levels) resulted in a significant increase in the odds of MetS [21]. Previous studies examining the effect of combined lifestyle factors on other metabolic abnormalities showed similar results. A lifestyle pattern consisting of a higher snack and dairy consumption and lower levels of physical activity was associated with obesity and unfavorable glycaemic indices, lipid profile and increased high-sensitivity C-reactive protein concentrations among urban Sri Lankan women [4]. In addition, a previous study by our group investigating the role of combined lifestyle factors on elevated blood pressure showed that a higher adherence to the ‘Fast food and smoking’ and the ‘Traditional and sedentary’ patterns increased its odds among Qatari women [6].

Looking at the diet profile of the ‘High Risk’ lifestyle pattern identified in this study, it shares considerable similarities with the Western dietary pattern, commonly reported in the literature, as higher consumption of fast foods, sweets and sweetened beverages and a lower consumption of whole grains [22]. The consumption of such energy-dense foods has been shown to alter lipoprotein kinetics and metabolism in MetS, inducing an unfavorable shift in markers of TG-rich lipoprotein, including increase apo-lipoprotein C-III, VLDL-apo B and remnant like particles rich in cholesterol and TG [23]. Furthermore, the lower intakes of whole grain products prevalent in the western type of diets was also reported to be associated with a lower intakes of fiber and magnesium both of which have been implicated in protecting against MetS [24]. Sugar sweetened beverages, also part of the ‘High Risk’ pattern identified in this study, were previously found to be associated with lower satiety compared with carbohydrates consumed in solid form [25]. Recently, compelling evidence supported the detrimental effect of these beverages on the risk of obesity and the MetS [26]. This association was explained by the fact that consumption of high glycemic index foods such as sweets and sugar sweetened beverages is characterized by fast-release of carbohydrate, a higher blood glucose levels, greater insulin demand, resulting in a self-perpetuating insulin resistance state, the latter being directly linked to the MetS [27].

However, although adherence to the Western dietary pattern or patterns with similar constituents should increase the risk of MetS, the large body of studies which examined this association produced inconsistent results. For instance, In Italy, no significantly association was found between the ‘Unhealthy’ dietary pattern and the risk of MetS in a cohort of healthy adults [28]. Similarly, in Korean women, the ‘Western’ dietary pattern was not associated with MetS [29]. Moreover, among women of the Framingham Offspring/Spouse cohort, a null association was found between dietary patterns, including the Higher Fat and the Empty Calorie, with MetS [30]. In other studies, positive associations were reported between the ‘Western’ and western like dietary patterns and MetS. Adherence to the Fast Food/Dessert pattern increased the odds of MetS in a national sample of Lebanese adults, whereby participants in the highest quintile of that pattern had three times higher odds of MetS as compared to those in the first quintile [31]. A possible explanation of these inconsistencies is the potential and cumulative interaction between the different lifestyle factors, including diet physical activity and smoking and their possible synergistic effect on the odds of MetS. In fact, combining diet and exercise programs have been shown to reduce rates of MetS development and its components [32]. Together with an exercise regimen, both high- and low-glycemic diets (non-energy restricted) had similar effect in reducing blood pressure, TG, and glucose in MetS [33]. Such an exercise regimen appears to have helped in lowering visceral fat and enhancing insulin sensitivity [34,35]. In addition to diet and physical activity, tobacco use has been implicated in the etiology of MetS due to its effects on WC, blood lipids and blood pressure [36]. Studies investigating the association between smoking and the risk of MetS, however, have frequently led to inconsistent results [37,38,39]. In their commentary Rabeus *et al*. argue that such inconsistent associations between smoking and the MetS could be due to the presence of other lifestyle components prevalent in smokers. For example, physical inactivity and alcohol drinking are known to be more often present in smokers as compared to non-smokers. The authors hence recommended the examination of smoking as part of a lifestyle pattern rather than a single exposure in relation to MetS [40]. In this study, alcohol, though important, was not included as a lifestyle characteristic because it is not considered part of the Qatari lifestyle. In fact, the religion of Islam, which plays a strong role in the Qatari way of life, discourages the consumption and trade of alcoholic beverages [41].

Hence, the findings of this study further confirmed a synergistic effect between diet, physical activity and smoking and showed that a ‘High Risk’ lifestyle pattern consisting of an ‘unhealthy’ type of diet combined with a low level of physical activity and smoking leads to a statistically significant increase in the odds of MetS.

The lack of association between the Prudent and Traditional lifestyle patterns with MetS could be explained by the combinations of factors characterizing these patterns. For instance, though the ‘Prudent’ pattern was driven by dietary factors that are postulated to have a favorable effect on health, such fruits, vegetables and fish [42], it had no protective effect against the odds of MetS, possibly because of variability of physical activity levels and smoking prevalence within this pattern. The ‘Traditional’ pattern, on the other hand, had a mix of factors with opposite effects on MetS. For instance, this pattern included dietary factors such as refined grains in addition to fruits and natural juices. It was also characterized by a lower smoking prevalence as well as lower physical activity levels (Table 2). Hence the findings of this study add evidence that a single behavioral factor (diet, physical activity and smoking) may not show an effect on the risk of MetS unless present within a combination of factors, which together exert a synergistic effect and lead to a significant effect on disease risk.

In addition to investigating lifestyle patterns and their association with MetS in the study population, the findings of this study provided a national estimate of the prevalence of MetS and its components among Qatari women of childbearing age. Such a prevalence of the MetS (16%) is comparable to other estimates for women of similar age group from neighboring countries such as Jordan (5.3% in 20–29 years, and 17.1% in 30 to 39 years) [43], Iran (6.9% in 20–29 years and 15% in 30 to 39 years) [44], and Turkey (15.09% in premenopausal women) [45]. Our findings also indicated that, among the components of MetS, elevated WC was the most prevalent risk factor (48.5%). Such a high prevalence is alarming, particularly in light of accumulating evidence for the role of abdominal obesity in cardiovascular diseases. Abdominal obesity results in a pro-inflammatory state with increased visceral fat deposits and alteration of adipokine secretion, with concomitant insulin resistance, alterations of nitric oxide/superoxide balance and endothelial dysfunction [46].

This study had several limitations. First, the findings of this study could not infer a causal relationship given the cross-sectional design. However, in order to reduce possible reverse causation, participants who reported diagnosis with a chronic condition which may have affected their dietary habits were excluded in the analysis of lifestyle patterns and their association with MetS. Second, the use of PCFA for lifestyle patterns derivation requires several assumptions related mainly to the number of retained factors and their labels. To minimize subjectivity, the selection of the factor solution was done after evaluating standard criteria such as the scree plots and eigenvalues [47]. Third, the questionnaire for dietary intake used in this study did not include information on portion size. However, estimating the portion size of every food consumed requires a complex cognitive process that may overburden the participants, especially that different portion sizes of the same food could be consumed over various meals [48]. Furthermore, in many instances people are not aware of the portion size of the food they are eating [49]. Taken together, these limitations of estimating portion size may lead to errors in data reporting and to data omission and hence would not contribute significantly beyond frequency data in improving the accuracy of dietary assessment [50]. Recently, nutrition research aimed to rank individuals based on their dietary intake of specific foods or dietary patterns (rather than determining the absolute intake) using non-quantitative dietary intake assessment tools, with a lesser focus on the size of portion consumed [51,52]. Fourth, as noted in most questionnaires-based data collection, the interview approach could have incurred a social desirability bias. Survey participants could have answered in a way that they perceive as acceptable or favorable to the interviewer [53]. In this study, field workers received extensive training to reduce judgmental verbal and non-verbal communication during data collection in order to minimize any social desirability bias. Lastly, it remains important to note that, although alcohol consumption is an important lifestyle factor, it was not considered part of the Qatari lifestyle and hence no information about alcohol consumption was collected [41].

## 4. Materials and Methods

### 4.1. Study Design

Data for this study were drawn from the Qatar National STEPwise Survey conducted in 2012 on a nationally representative sample of Qatari adults (18–64 years old). For the survey design, including sampling and data collection, the guidelines of the WHO STEPwise approach to non-communicable disease risk factor surveillance were followed [54]. Households were randomly selected using multi-stage cluster sampling. Each cluster consisted of 60–70 contiguous blocks and was selected using probability proportional to size sampling. Qatar has seven main municipalities: Doha, Al Rayyan, Al Warka, Umm Salal, Al Khor, Al Shamal, and Al Daayeen. In each of these municipalities, a total of 95 clusters were selected. Within each cluster, 30 households were randomly identified [55]. In the household, one adult was selected to participate. Eligibility criteria were (1) Qatari nationality and (2) aged between 18 and 64 years. In case more than one subject was eligible, only one adult was randomly selected using the Kish method [56]. Of the 2850 households that were approached, 2496 agreed to participate in the study. Hence the rejection rate was 12%. The Ministry of Public Health and the Ministry of Development Planning and Statistics, Doha, Qatar reviewed the survey protocol and granted it ethical approval. All subjects gave informed consent for their participation. Further details about the design and protocol of the survey are presented in the Qatar STEP wise report (2012) [57].

For the purpose of this study, survey data of women of child bearing age (18–45 years), and not pregnant at the time of the interview were included. Out of 916 women who met the inclusion criteria, 584 had complete biochemical analysis’ results (63.7%) and their data were used to estimate the prevalence of MetS and its components in the study population. For the derivation of lifestyle factors and later the association of these factors with MetS and its components, subjects were excluded if they had a known diagnosis of hypertension, diabetes or any other health condition that may affect their dietary intake (*n* = 99). Furthermore, 67 subjects had missing or incomplete dietary intake, lifestyle characteristics, or anthropometric measurements data and were also excluded. A comparison of survey participants who were included in the lifestyle pattern derivation (*n* = 418) and those excluded due to incomplete and/or missing data (*n* = 485) showed no significant differences according to all sociodemographic characteristics (data not shown).

### 4.2. Data Collection

Data collection took place during face-to-face interviews at the participants’ home by trained field workers. The three steps included within the WHO STEPwise approach manual were followed in data collection as follows:

Step 1: A multicomponent questionnaire consisting of sections related to sociodemographic, lifestyle characteristics and dietary intake and meal pattern was used. The sociodemographic section included questions about age, education, job type, marital status, parental consanguinity, family history of diabetes and hypertension. Lifestyle characteristics related to the number of meals not prepared at home, smoking and physical activity were examined. The following question was used to assess ‘meals not prepared at home’: On average, how many meals per week do you eat that were not prepared at home? (by meals I mean breakfast, lunch and dinner). Regarding smoking, questions about smoking status (nonsmoker, current or past smoker) and exposure to passive smoking (number of days/week) were included in the questionnaire. Physical activity was assessed using the WHO standard questionnaire (Arabic version) whereby the intensity, duration, and frequency of each activity are accounted for [58]. Physical activity was further characterized as low, moderate, or high based on total METS-min per week [18,59]. The dietary intake of survey participants was evaluated using a non-quantitative food frequency questionnaire (FFQ). In this questionnaire, the portion size of the food consumed was not assessed. The FFQ consisted of 13 food groups, namely refined grains, whole grains, fruits, vegetables, beans, milk and dairy products, meat, poultry, fish and sea food, sweets, sweetened beverages, fast foods, and natural juices. Examples of food items under each group were provided to facilitate the participants’ recall of her dietary intake. Consumption frequency was recorded as number of days per week the food/food group was consumed. The non-quantitative FFQs, by providing information on the usual intake of a particular food/food group, are increasingly acknowledged as valuable tools to determine dietary patterns and trends at the population level [60]. Before initiation of data collection, pilot testing of the various components of the questionnaire was conducted on a sample of Qatari adults in order to determine their clarity and their cultural sensitivity. The results of the pilot testing were not included in this analysis. Subjects were asked to make reference to a typical week when answering the physical activity and the dietary intake questionnaires. In addition, drug treatment prescribed by a doctor or other health worker was assessed for the following conditions: hypertension, dyslipidemia, and elevated blood glucose. The reference period adopted for assessing the subjects’ medication use was the past two weeks.

Step 2: Blood pressure and anthropometric measurements including height, weight, and WC of participant. Blood pressure was measured using an Omron M7 sphygmomanometer (Omron BP785; Shanghai, China). For each of the systolic and diastolic blood pressure, three readings were obtained within five minutes intervals. The means of the second and the third readings were computed and used in the study. Weight and height were measured using calibrated equipment and standardized techniques. Subjects were dressed in light indoor clothing and were asked to remove their shoes. Weight was recorded to the nearest 0.1 kg. For the height measurement, a stadiometer was used while subjects had their shoes off. The height reading was recorded to the nearest 0.5 cm. The Body mass index (BMI) was computed using the following formula: BMI = Weight(kg)/(Height(m))^2^. Using a calibrated plastic measuring tape, waist circumference (WC) was measured with the subject in a standing position. The tape was positioned at equal distance between the bottom of the rib cage and the top of the iliac crest. Measurements were taken twice, to the nearest 0.1 cm, and the average of the 2 values was adopted.

Step 3: Biochemical measurements were conducted using dry chemistry equipment and supplies and included fasting blood glucose, TG and HDL. All tests were carried out after a 12-h fast. The Professional CardioCheck PA (Polymer Technology Systems, Inc., Indianapolis, IN, USA), as recommended by the WHO, was used for the analysis of the biomarkers. Previous research examining the accuracy of the CardioCheck showed a correlation of its results with those of laboratory standard techniques of 0.95 for HDL (CV = 5%–10%) and 0.98 for triglycerides (CV ≈ 14%) [61].

### 4.3. Definition of MetS

MetS was defined by the ATPIII criteria, whereby MetS is present if a woman had three or more of the following five criteria: WC over 88 cm, a blood pressure greater or equal to 130/85 mmHg (or taking antihypertensive drug when the patient was previously diagnosed with hypertension), fasting triglyceride (TG) level over 150 mg/dL (Or on drug treatment for elevated triglycerides), fasting high-density lipoprotein (HDL) cholesterol level less 50 mg/dL (Or on drug treatment for reduced HDL-C) and fasting blood sugar ≥100 mg/dL (or on drug treatment for elevated glucose) [19].

### 4.4. Lifestyle Patterns Derivation

For the lifestyle pattern derivation, variables corresponding to dietary intake (13 foods/food groups), physical activity (number of MET-min) and smoking (non-smoker, past smoker and current smoker) were included in the exploratory Principal Component Factor Analysis (PCFA). Prior to running the PCFA, a few diagnostics were examined including the correlation matrix between all the variables, The Kaiser-Meyer-Olkin test (>0.7) and the chi square for Bartlett test of sphericity (*p* < 0.05). The PCFA was implemented using an orthogonal rotation (Varimax) of factors; in which the axes are maintained in 90 degrees. To decide on the number of factors to be retained, three criteria were taken into consideration (1) the Kaiser criterion which states that a factor to be retained ought to have an eigenvalue greater than 1; (2) the inflection point or the ‘elbow’ of the scree plot (3) and the relevance and interpretability of the factors to be retained. The least squares regression method was used for the calculation of the factor scores (with a mean of zero). Each participant had a score on each of the three factors. Factor scores reflected each individual’s placement on the factors and consequently her adherence to the identified pattern. Factor loadings indicated the strength and direction of the association between the patterns and the lifestyle variables used in the PCFA. For each pattern, participants were grouped into tertiles of pattern scores. For the labelling of the patterns, lifestyle variables with a rotated factor score greater than 0.3 were considered [62]. When a variable loaded high (>0.3) on more than one factor, the factor with the highest loading was considered for factor labeling.

### 4.5. Statistical Analyses

The study sample distribution across age and geographical area in Qatar was different than that of the Qatar census data as provided by the Ministry of Development Planning and Statistics (Qatar Statistics Authority, Doha, Qatar) (2010) [55]. Hence, sampling weights were applied in order to correct for the selection probabilities. The weights were calculated as the inverse of the sampling fractions. Descriptive statistics including proportions and means ± standard deviations were used for categorical and continuous variables respectively. *t*-Test, chi-square test, and ANOVA were used to chart comparisons between groups with and without MetS. Multivariate logistic regression models were used to evaluate the associations between the lifestyle patterns and MetS, whereby MetS was set as the outcome variables and tertiles of lifestyle patterns’ scores were set as independent variables. Pattern scores were grouped into tertiles based on their distribution. Subjects who belonged to the third tertile of a pattern’s score had the highest adherence to this pattern as compared to those who belonged to the first and second tertiles. Multivariate adjustment was applied to the logistic regression models. Variables were adjusted for if they were associated with either MetS or the lifestyle patterns. Such variables included age, education, marital status, job type, parental consanguinity, family history of diabetes, family history of high blood pressure, passive smoking, and number of meals not prepared at home. All analyses were two tailed and a *p*-value <0.05 was considered statistically significant. The Statistical Package for the Social Sciences (SPSS; version 14.1) was used for all computations [63].

## 5. Conclusions

The present study identified three main lifestyle patterns amongst Qatari women of childbearing age: the ‘High Risk’, the ‘Prudent’ and the ‘Traditional’ patterns. The ‘High Risk’ consisting of a western-like diet, low levels of physical activity and a higher prevalence of smoking was associated with more than doubling the odds of MetS in the study population. These findings support the combined effect of lifestyle behaviors in relation to diseases with complex and multifactorial etiologies such as the MetS. It also provided the necessary evidence for health authorities in Qatar to focus on ‘holistic’ lifestyle patterns modifications in the development of culturally sensitive strategies and interventions targeted at disease prevention among women of childbearing age. Such evidence-based interventions are of major public health importance particularly in light of the deleterious effect of MetS on both mother and child’s health in predisposing to higher risk of gestational diabetes, cardiovascular diseases and diseases later in life. Public health policy may encourage the integration of lifestyle intervention in the healthcare systems; interventions that are rooted in the community, its culture and its values. More studies are warranted to confirm these findings in other populations.

## Figures and Tables

**Table 1:** Socio-demographics, lifestyle and anthropometric characteristics of study participants and their association with MetS (*n* = 418).

	Total *n* = 418	Women without MetS *n* = 376	Women with MetS *n* = 42	OR (95% CI) ^a^
Age (years)	30.89 ± 6.96	30.50 ± 7.02	34.41 ± 5.26	1.09 (1.04–1.14) *
18–24	72(17.2)	70(18.6)	2(4.9)	-
25–34	217(51.9)	197(52.3)	20(48.8)	3.37 (0.80-14.15)
35–45	129(30.9)	110(29.2)	19(46.3)	5.83 (1.38-24.67) *
Education				
Up to intermediate level	72(17.3)	63(16.8)	9(22.0)	-
Finished high school	154(37.0)	144(38.4)	10(24.4)	0.48 (0.19–1.22)
University/graduate level	190(45.7)	168(44.8)	22(53.7)	0.90 (0.40–2.05)
Marital status				
Not married	149(35.7)	140(37.2)	9(22.0)	-
Married	268(64.3)	236(62.8)	32(78.0)	0.49 (0.23–1.05)
Job type				
Housewife	125(29.9)	110(29.3)	15(35.7)	-
Not working	77(18.4)	71(18.9)	6(14.3)	0.59 (0.21–1.62)
Non-governmental employee	17(4.1)	15(4.0)	2(4.8)	1.09 (0.24–4.90)
Governmental employee	199(47.6)	180(47.9)	19(45.2)	0.78 (0.38–1.61)
Parental consanguinity				
No	288(68.9)	259(68.9)	29(69.0)	-
Yes	130(31.1)	117(31.1)	13(31.0)	0.99 (0.50–1.99)
Family history of diabetes				
No	124(29.7)	117(31.1)	7(16.7)	-
Yes	294(70.3)	259(68.9)	35(83.3)	2.45 (1.03–5.82) *
Family history of high blood pressure				
No	143(34.2)	129(34.3)	14(33.3)	-
Yes	275(65.8)	247(65.7)	28(66.7)	1.05 (0.53–2.07)
Number of meals not eaten at home (per week)	2.48 ± 2.25	2.50 ± 2.27	2.24±2.10	0.95 (0.81–1.10)
Smoking status				
Nonsmoker or past smoker	408(97.8)	370(98.4)	38(92.7)	-
Current smoker	9(2.2)	6(1.6)	3(7.3)	5.46 (1.40–21.25) *
Exposure to passive smoking (days/week)	1.28 ± 3.10	1.29 ± 3.14	1.19 ± 2.76	0.99 (0.89–1.10)
Total physical activity (Met-minutes per day)	420 ± 779	415 ± 773	466 ± 836	1 (1.0–1.0)
Physical activity level ^b^				
Low	227(54.2)	207(55.1)	20(46.5)	-
Moderate	89(21.2)	78(20.7)	11(25.6)	1.42 (0.64–3.13)
High	103(24.6)	91(24.2)	12(27.9)	1.34 (0.62–2.89)
Body mass index (kg/m^2^)	29.03 ± 7.52	28.29 ± 7.27	35.70 ± 6.48	1.11 (1.07–1.15) **
Obese(≥30 kg/m^2^)	151(36.1)	116(30.9)	35(83.3)	10.97 (4.75–25.33) **

^a^ OR(s) are derived from bivariate logistic regression; ^b^ The moderate and high levels of physical activity were assigned as per Bauman, A., *et al*. [18]: Moderate category if a minimum of one of the following three criteria is met: (a) Three days of vigorous activity of at least 20 min per day; (b) 5 days of moderate-intensity activity or walking for more than 30 min per day with each activity lasting for more than 10 min each time; or (c) 5 days of any combination of activities totaling to a minimum of 600 MET-minutes/week; High category if one of the following two criteria is met: (a) Vigorous-intensity activity on more than 3 days/week and totaling to a minimum of 1500 MET-minutes/week; or (b) more than 5 days of any combination of activities achieving a minimum of 3000 MET-minutes/week; Low category if none of the criteria for the Moderate or the High categories apply. * Significant at *p* < 0.05, ** significant at *p* < 0.001.

**Table 2:** Factor loading matrix of the three identified lifestyle patterns among a nationally representative sample of Qatari women ^a^ (*n* = 418).

	Lifestyle Patterns		
	High Risk	Prudent	Traditional
Fast foods	0.63		
Sweetened beverages	0.58		
Whole grains	−0.51	0.30	
Sweets	0.51		
Refined grains	0.49	−0.22	0.33
Poultry	0.47		
Physical activity	−0.36		
Fruits		0.71	
Vegetables		0.65	
Natural juices		0.62	
Fish and sea food		0.57	
Beans			0.70
Meats			0.62
Milk and milk products			0.44
Smoking	0.26		−0.35
Percent variance explained	12.59	12.51	9.12

^a^ Factor loadings of less than 0.2 were not listed in the table for simplicity.

**Table 3:** Dietary intake, smoking, and physical activity of study participants by tertiles of scores corresponding to the three identified lifestyle pattern ^a^ ^b^ (*n* = 418).

	High Risk Pattern		Prudent Pattern		Traditional Pattern	
	1st Tertile	3rd Tertile	1st Tertile	3rd Tertile	1st Tertile	3rd Tertile
Lifestyle characteristics and dietary intake	n(%) or mean ± SD					
Fast foods (days/week)	0.67 ± 1.01	3.78 ± 2.3 **	2.04 ± 2.06	2.09 ± 2.18	2.09 ± 2.16	2.26 ± 2.22
Sweetened beverages (days/week)	0.76 ± 1.22	4.86 ± 2.65 **	2.92 ± 2.81	1.93 ± 2.4 **	2.6 ± 2.96	2.72 ± 2.73
Whole grains(days/week)	3.51 ± 2.96	0.65 ± 1.39 **	1.17 ± 2.2	2.75 ± 2.82 **	2.08 ± 2.73	1.6 ± 2.38
Sweets (days/week)	2.5 ± 2.21	5.66 ± 2.07 **	3.98 ± 2.67	4.16 ± 2.59	4.32 ± 2.65	4.34 ± 2.57
Refined grains (days/week)	3.59 ± 2.71	6.41 ± 1.43 **	5.88 ± 2.05	4.56 ± 2.62 **	3.92 ± 2.73	6.12 ± 1.79 **
Poultry (days/week)	3.81 ± 2.06	5.8 ± 1.74 **	5.36 ± 2.07	4.7 ± 1.99 *	5.15 ± 2.18	5.33 ± 1.9
Total physical activity (Met-minutes per day)	558 ± 1063	323 ± 558*	455 ± 780	393 ± 639	566 ± 953	480 ± 729 *
Fruits (days/week)	3.74 ± 2.77	2.56 ± 2.49 **	1.01 ± 1.19	5.2 ± 2.23 **	2.43 ± 2.51	3.58 ± 2.57 **
Vegetables (days/week)	5.46 ± 2.38	5.06 ± 2.55	3.26 ± 2.52	6.79 ± 0.89 **	5.15 ± 2.51	5.26 ± 2.58
Natural juices (days/week)	3.83 ± 2.64	3.51 ± 2.56	1.83 ± 1.97	5.57 ± 2.19 **	3.31 ± 2.78	4.1 ± 2.72 *
Fish and sea food(days/week)	1.86 ± 1.5	1.3 ± 1.12 *	0.83 ± 0.71	2.41 ± 1.56 **	1.78 ± 1.63	1.44 ± 1.23 *
Beans (days/week)	1.55 ± 1.49	1.79 ± 1.79	1.42 ± 1.68	2.06 ± 1.67 *	0.67 ± 0.84	2.89 ± 2.04 **
Meats (days/week)	1.49 ± 1.38	2.03 ± 1.74 *	1.88 ± 1.87	1.81 ± 1.36	0.88 ± 0.89	3.04 ± 1.91 **
Milk and milk products(days/week)	5.82 ± 2.01	5.9 ± 2.07	5.85 ± 2.18	5.93 ± 1.98	4.86 ± 2.63	6.53 ± 1.32 **
Smoking						
Nonsmoker or past smoker	131(100)	144(94.7) *	130(97.0)	149(98.7)	127(92.7)	155(100) **
Current smoker	0(0.0)	8(5.3)	4(3.0)	2(1.3)	10(7.3)	0(0.0)

* *p* ≤ 0.05, ** *p* ≤ 0.001, ^a^ Intake of the various food groups referred to frequency of consumption as expressed by number of days per week the food/food group was consumed; ^b^ Significance was derived from ANOVA test for continuous variables and chi square test for categorical variables.

**Table 4:** Odds of the metabolic abnormalities and MetS across tertiles of factor scores assessed by multivariate logistic regression ^a^ in the study population (*n* = 418).

	Elevated WC ^b^	Hyper Triglyceridemia ^c^	Low HDL-C ^d^	Elevated Blood Pressure ^e^	Hyperglycemia ^f^	MetS ^g^
Lifestyle Patterns						
**High risk**						
1st tertile	1	1	1	1	1	1
2nd tertile	0.99 (0.58–1.69)	0.71 (0.33–1.55)	1.30 (0.77–2.20)	0.91 (0.40–2.07)	1.59 (0.75–3.38)	1.06 (0.41–2.75)
3rd tertile	0.94 (0.55–1.63)	0.68 (0.30–1.54)	1.19 (0.70–2.04)	1.96 (1.02–4.70)	1.72 (0.80–3.68)	2.47 (1.04–5.39)
**Prudent**						
1st tertile	1	1	1	1	1	1
2nd tertile	1.52 (0.89–2.67)	0.89 (0.39–2.05)	0.99 (0.59–1.67)	1.72 (0.784–3.806)	0.61 (0.28–1.32)	1.69 (0.65–4.39)
3rd tertile	1.03 (0.60–1.77)	0.81 (0.36–1.81)	0.75 (0.44–1.27)	1.33 (0.596–2.989)	1.08 (0.53–2.19)	2.05 (0.81–5.15)
**Traditional**						
1st tertile	1	1	1	1	1	1
2nd tertile	0.80 (0.47–1.37)	1.55 (0.71–3.41)	0.89 (0.52–1.52)	1.35 (0.62–2.92)	1.64 (0.81–3.34)	1.16 (0.49–2.73)
3rd tertile	1.45 (0.85–2.46)	0.84 (0.37–1.91)	1.67 (0.97–2.68)	1.51 (0.71–3.21)	1.11 (0.52–2.36)	1.13 (0.49–2.63)

^a^ Values presented in the table are OR and 95% CI. ORs were adjusted for age, education, marital status, job type, parental consanguinity, family history of diabetes, family history of high blood pressure, passive smoking, number of meals not eaten at home; ^b^ Elevated Waist circumference (≥88 cm); ^c^ High Fasting Triglycerides: ≥150 mg/dL; ^d^ Low HDL(<50 mg/dL); ^e^ High Blood Pressure: SBP ≥ 130 mmhg or DBP ≥ 85 mmhg; ^f^ hyperglycemia:fasting blood glucose ≥100 mg/dL; ^g^ MetS was diagnosed if a woman had three or more of the following five criteria: WC over 88 cm, blood pressure ≥ 130/85 mmHg (or on antihypertensive drug treatment in a patient with a history of hypertension), fasting triglyceride (TG) level over 150 mg/dL (Or on drug treatment for elevated triglycerides), fasting high-density lipoprotein (HDL) cholesterol level less 50 mg/dL (Or on drug treatment for reduced HDL-C) and fasting blood sugar ≥100 mg/dL (or on drug treatment for elevated glucose) [19].

## Abbreviations

NCEP ATPIII	The National Cholesterol Education Program’s Adult Treatment Panel III
BMI	Body Mass Index
CI	Confidence Interval
CV	Coefficient of Variation
FFQ	Food Frequency Questionnaire
GCC	Gulf Cooperation Council
GDP	Gross Domestic Product
GPAQ	Global Physical Activity Questionnaire
HDL	High Density Lipoprotein
MET	Metabolic Equivalent of Task
MetS	Metabolic Syndrome
NCD	Non communicable diseases
OR	Odds Ratios
PCFA	Principal Component Factor Analysis
SPSS	Statistical package for Social Sciences
TG	Triglyceride
WC	Waist Circumference
WHO	World Health Organization
